# Body and Mind Intertwined: Physical Activity Intensity, Sleep Quality, and Depressive Symptoms: Cross-Sectional Associations and Exploratory Path Analysis

**DOI:** 10.3390/jcm15145754

**Published:** 2026-07-22

**Authors:** Witold Wit Hryniewicz, Igor Barczak, Kacper Fręśko, Zuzanna Szarzyńska, Hubert Węclewski, Jakub Żubrowski, Barbara Cybula, Bartosz Dmuchowski, Agnieszka Pluto-Prądzyńska

**Affiliations:** 1 Faculty of Medicine, Poznan University of Medical Sciences, 61-701 Poznan, Poland; hryniewiczwitek@wp.pl (W.W.H.); zuzanna.szarzynska54@gmail.com (Z.S.);; 2 The Student Scientific Society, Poznan University of Medical Sciences, 60-806 Poznan, Poland; 3 Department of Immunology, Chair of Pathomorphology and Clinical Immunology, Poznań University of Medical Sciences, 60-806 Poznan, Poland; 4 Faculty of Medicine, Medical University of Lodz, T. Kościuszki 4, 90-419 Lodz, Poland; 5 Lifestyle Medicine Lab, Chair of Pathomorphology and Clinical Immunology, Poznań University of Medical Sciences, 60-806 Poznan, Poland

**Keywords:** depression, sleep, physical activity

## Abstract

**Background**: The alarming trend of rising depression and insomnia among the world’s population, especially in young adults, demands urgent attention from both clinical and lifestyle medicine perspectives. This study aimed to evaluate whether habitual physical activity (PA) could improve sleep quality and depressive symptoms. It explores whether sleep parameters mediate the relationship between training intensity and depression. **Methods**: This cross-sectional study examined the relationships between PA, sleep quality, and depression in 349 participants, predominantly young adults, aged 16–54 (Median: 21, Q1–Q3: 20–23). Depressive symptoms and sleep quality were assessed using the Beck Depression Inventory (BDI) and Pittsburgh Sleep Quality Index (PSQI). PA parameters, including training intensity and frequency, were evaluated via a study-specific self-report tool. Mediation effects were assessed utilising Structural Equation Modelling (SEM) to evaluate direct, indirect, and total effects. **Results**: Higher training intensity and training volume were associated with lower depressive symptoms. Except for the daytime dysfunction domain, the current data did not support sleep as a mediator in the relationship between physical activity and depressive symptoms. Participants with 3–5 years of training experience exhibited lower BDI scores compared to those with less than 12 months of experience. Interestingly, the type of PA did not influence BDI or PSQI scores, suggesting that consistency and intensity are more critical than the kind of exercise itself. **Conclusions**: Systematic, high-intensity PA, especially for a prolonged period of time, is significantly associated with lower depressive symptoms. The exploratory nature of this study highlights training frequency and intensity as potential factors that need further research. These findings highlight the mind–body connection and demonstrate that PA might be an intervention supporting mental well-being.

## 1. Introduction

Depression is a disorder characterised by mood changes and physical symptoms lasting at least two weeks. In recent years, there has been a global increase in the incidence of depression, making it a global problem that poses a significant socioeconomic challenge due to both its prevalence and the increased risk of other diseases [1]. Globally, depression is one of the leading causes of disability among people aged 15–29, with students constituting a group at increased risk for this disorder [2]. Although it is not limited to young people, they are a demographic group particularly affected by it. According to current research, depression statistically affects women more often than men; furthermore, people with lower socioeconomic status are more likely to develop it. Among students, lifestyle and health-related factors such as regular PA, smoking, stress and sleep quality have a greater association with the severity of depressive symptoms than gender [3]. In today’s fast-paced world, more and more young people do not have time to obtain enough sleep, prepare healthy meals or exercise regularly.

Depression and insomnia are interrelated and influence each other [4]. According to epidemiological data, people struggling with insomnia are five times more likely to develop anxiety disorders and depression than those without sleep disorders. At the same time, over 90% of people with depression report a decline in sleep quality, creating a bidirectional cause-and-effect cycle that severely undermines mental well-being [4]. Insomnia, like depression, affects women more often than men [5]. While these trends are well-documented in community and student cohorts, anchoring this argument in clinical population data provides a critical counterpoint. In a recent study analysing a real-world outpatient cohort using a validated insomnia measure, the Insomnia Severity Index, clinically significant insomnia was found to be highly prevalent, affecting 40.5% of psychiatric outpatients. Crucially, insomnia severity scores were highest precisely among individuals with depressive disorders, a finding that strongly reinforces the comorbidity argument and highlights the transdiagnostic burden of sleep disturbances [6]. This issue is further exacerbated by contemporary environmental factors; research indicates that excessive exposure to blue light from electronic device screens disrupts melatonin production, which negatively affects the sleep–wake cycle, thereby reducing sleep quality [3].

In response to these growing public health challenges, lifestyle medicine has emerged as a crucial framework for preventative and therapeutic intervention. Driven by social media, lifestyle medicine has become a particularly popular topic in recent years. It is based on six main pillars: a healthy diet, PA, stress management, sleep, avoiding substance use, and fostering social connections [7]. These pillars interact with one another, both positively and negatively; therefore, it is extremely important to strive to strengthen each of them, as this yields the best health outcomes [8]. In this article, we focus on the link between two of these six pillars: sleep and PA.

Utilising PA as a holistic intervention holds significant promise for managing depressive disorders. Various methods are used to treat depression, such as pharmacotherapy and psychotherapy, but these are not always sufficient for the individual. Taking a holistic approach to the individual, incorporating regular PA into the overall management plan is a promising option [9]. It has been demonstrated that the effectiveness of PA is comparable to that of pharmacological treatment and psychotherapy; however, given its accessibility and cost-effectiveness, it could be considered a first-line intervention [9]. Even a small amount of aerobic activity is associated with a reduction in depressive symptoms, which is particularly important for individuals with multiple comorbidities, attenuated mood, and consequently often limited physical fitness [10]. Aerobic exercises supervised by an experienced instructor or performed in groups have proven to be the most effective, suggesting that the social aspect, which directly taps into another pillar of lifestyle medicine, also plays an important role in mitigating symptoms of depression [9]. In studies, the best results were achieved by individuals at an early school age and in the perinatal period [9]. Additionally, participants’ planning of PA and self-monitoring also contribute to better outcomes [11].

Beyond its direct psychological benefits, the impact of PA extends to sleep regulation, further reinforcing its antidepressant effects. Insomnia is the most commonly reported sleep-related problem, with approximately 20% of the adult population experiencing its symptoms [12,13]. Since sleep quality has a significant impact on mental health, studies have shown that PA helps improve sleep quality. It has been shown to reduce stress and low mood while improving sleep quality. At the same time, better sleep after exercise also has an anti-anxiety effect, establishing a positive feedback loop [13]. On a neurological level, PA modulates the brain; it increases neuroplasticity and improves function in areas responsible for sleep, such as the motor cortex, prefrontal cortex and hippocampus [12].

While such non-pharmacological options are frequently discussed in the context of older populations to avoid polypharmacy, understanding these relationships is equally critical for young adults and students [14]. This younger demographic faces unique academic stressors, lifestyle disruptions, and a high incidence of concurrent sleep and mood disturbances. Identifying accessible, lifestyle-based strategies is therefore essential to support mental well-being and sleep hygiene early in life, potentially preventing the escalation of these symptoms.

However, to maximise these potential benefits, the specific characteristics of the exercise must be carefully evaluated rather than lumping all activities together. Early studies on the impact of PA on mental health and sleep tended to lump all types of activity together. Today, it is considered essential to differentiate between specific types of training, given the distinct biomechanical and physiological mechanisms underlying each form. It is important to note that not every type of PA will be beneficial for every individual. When selecting the right exercise regimen, it must be tailored to the individual based on several key factors such as intensity, age, gender, frequency, underlying medical conditions, and personal preferences. This personalised approach ensures that individuals reap the maximum benefits from movement while simultaneously reducing the likelihood that they will lose motivation and abandon their exercise routine [15].

Based on the existing literature and the clear need to understand how distinct exercise parameters co-occur with these variables, the main aim of this study was to investigate which characteristics of PA were most strongly associated with sleep quality and the severity of depressive symptoms. Specifically, this study utilised an exploratory path analysis to investigate three specific endpoints:

1. Determining whether the intensity of PA is directly associated with depressive symptoms, as well as indirectly through an exploratory statistical pathway involving sleep quality;
2. Examining the statistical relationships between the type of PA, sleep quality, and the severity of depressive symptoms;
3. Analysing how the number of weekly workouts relates to both sleep parameters and the severity of depressive symptoms.

Additionally, the study evaluated the cross-sectional associations of late caffeine consumption, cool-down sessions, and evening workout timing with sleep.

## 2. Materials and Methods

### 2.1. Data Collection Instruments

#### 2.1.1. Beck Depression Inventory

Depressive symptoms were assessed using the Beck Depression Inventory (BDI), one of the most widely used and validated self-report instruments for measuring the severity of depressive symptoms. The BDI consists of 21 items, each rated on a 4-point scale (0.3), yielding a total score ranging from 0 to 63 points. Higher scores indicate greater severity of depressive symptoms. Total scores were categorised as 0–8 (no or minimal depressive symptoms), 9–28 (moderate depressive symptoms) and 29–63 (severe depressive symptoms). The Polish-validated adaptation of the BDI by T Parnowski and W Jernajczyk was used in the present study [16]. BDI categories are used to describe symptom severity only and were not treated as clinical diagnoses.

#### 2.1.2. Pittsburgh Sleep Quality Index (PSQI)

Sleep quality was assessed using the Pittsburgh Sleep Quality Index (PSQI), a widely used and validated self-report instrument designed to evaluate subjective sleep quality over the preceding month. The PSQI consists of 19 individual items grouped into 7 domain scores, each rated from 0 to 3 points: (1) subjective sleep quality, (2) sleep latency, (3) sleep duration, (4) habitual sleep efficiency, (5) sleep disturbances, (6) use of sleeping medication, and (7) daytime dysfunction. The global PSQI score is the sum of all 7 domains and ranges from 0 to 21 points; higher scores indicate poorer sleep quality. A global score above 5 points is considered indicative of clinically poor sleep quality. The Polish validated adaptation of the PSQI by Badzio-Jagiełło et al. was used in the present study [17].

#### 2.1.3. Physical Activity Questionnaire

PA parameters were collected using a study-specific self-report questionnaire designed for the purposes of this survey, as no single validated instrument captures all the training-related dimensions relevant to this study. The questionnaire was not formally validated, and its items referred to habitual, typical training rather than to a fixed reference period. Participants reported the dominant type of PA they engaged in, which was subsequently classified into five categories: none, cardio, mixed, stretching, and strength training. The response options offered were strength, cardio, stretching, mixed, or “other” (free text); because a “none” option was not offered, the “no activity” category was derived from free-text answers indicating little or no exercise, together with a reported frequency of zero sessions per week. This is why the “no activity” group in the type variable (n = 21) matches the zero-session group in the frequency variable. Training frequency was reported as the number of training sessions per week and grouped into four categories: none, 1–2 times per week, 3–4 times per week, and 5 or more times per week. Training experience (i.e., the length of time the participant had been training) was reported in six categories ranging from less than 3 months to more than 10 years. Training intensity was self-rated on a 10-point scale from 1 to 10, with higher scores reflecting more intense training. Participants additionally reported whether they routinely performed a cool-down/relaxation session after training (never, sometimes, always), their habitual training schedule (morning, afternoon, evening, night, or a mixed schedule with a predominance of one of these), and their caffeine intake after 3:00 p.m. (none, 1 portion, or 2 or more portions; one portion was defined as 80 mg). All instruments were self-administered online in a single survey session.

### 2.2. Study Sample and Characteristics

This was a cross-sectional survey designed to examine associations between habitual PA, sleep quality and depressive symptoms. Data collection was conducted from January to February 2026, utilising a convenience sample recruited from the general population via an online questionnaire distributed through social media platforms, as well as printed flyers with QR codes/access links displayed at university premises, and through informal word-of-mouth referral. Recruitment was carried out mainly in a university setting, and the sample consequently consisted mostly of medical students. Participation was voluntary and anonymous. The initial database comprised 399 respondents. Prior to analysis, the following participants were excluded: those reporting current use of antidepressant and/or sleep-inducing medications (n = 48), one respondent who left the questionnaire entirely blank, and one respondent who reported an age of 13 years. The final analytic sample comprised 349 participants (median age 21 years, Q1–Q3 20–23, range 16–54). Beyond the instruments described above, the questionnaire also recorded physician-diagnosed chronic conditions, regular medication use, including psychiatric drugs, use of sleep- or recovery-oriented supplements, weekly alcohol consumption, and use of blue-light-emitting devices within one hour before sleep; these were used to characterise the sample and to define the exclusions above.

### 2.3. Statistical Analysis

All statistical analyses were performed using R software, version 4.6.0, with the lavaan package used for mediation analyses. Descriptive statistics, including means, standard deviations, medians, quartiles, and ranges, were calculated for all continuous variables. For categorical variables, absolute and relative frequencies (n and %) are reported.

Non-parametric tests were used for all group comparisons. Differences between two independent groups were assessed with the Mann–Whitney U test, while differences among three or more groups were evaluated using the Kruskal–Wallis test; where a significant overall effect was detected, pairwise post hoc comparisons were performed with Dunn’s test. Associations between continuous variables were examined using Spearman’s rank correlation coefficient.

Given the number of comparisons, the analyses are interpreted as exploratory. For the primary PA predictors, *p*-values were adjusted for multiple testing using the Benjamini–Hochberg false-discovery-rate (FDR) procedure, applied within families of related tests (the eight PSQI outcomes for each predictor, and the set of mediation models); both uncorrected and FDR-adjusted *p*-values are reported. The secondary analyses of cool-down behaviour, training schedule and post-3:00 p.m. caffeine intake are reported with uncorrected *p*-values and interpreted as exploratory only.

To determine whether sleep quality mediates the relationship between PA (operationalized as training intensity, a continuous variable) and depressive symptom severity, a series of mediation analyses were performed using structural equation modelling (SEM), also referred to as path analysis, with each of the seven PSQI domains and the global PSQI score tested separately as the mediating variable (M), training intensity as the independent variable (X), and the BDI score as the dependent variable (Y). The seven PSQI components (0–3) were treated as continuous indicators. Although these component scores are ordinal, parametric methods are robust to this feature, and for four-category scales, parametric (Pearson-based) and rank-based estimates yield virtually identical results, supporting their use as continuous indicators [18]. Bootstrap confidence intervals (95% CI) for the direct, indirect, and total effects were computed to assess the significance of mediation; specifically, raw (unstandardised) coefficients are reported with bias-corrected and accelerated (BCa) bootstrap confidence intervals based on 10,000 resamples, and missing data were handled with full-information maximum likelihood (FIML), so that all models share the same information base and the total effect is identical across the single-mediator models. Each model was estimated both unadjusted and adjusted for age, sex, BMI, and training experience, and it is reported whether the indirect effects persisted after adjustment. Because the data are cross-sectional, these models are presented as exploratory path analyses and do not establish temporal or causal mediation. For all analyses, the significance threshold was set at *p* < 0.05.

## 3. Results

### 3.1. Sample

The final analytic sample consisted of 349 participants. The sample included 215 women (61.60%), 133 men (38.11%), and 1 participant who did not disclose their sex (0.29%). The mean age was 23.06 years (*SD* = 6.50; *Me* = 21 years; *IQR* = 20–23; range: 16–54). The mean body mass index (BMI) was 22.44 kg/m^2^ (*SD* = 3.56; *Me* = 22.15 kg/m^2^; range: 15.74–40.14). Most participants were of normal weight (70.20%), while 13.75% were overweight, 9.74% were classified as thinness or underweight combined, and 4.30% were classified as obese (classes I-III combined, due to the small number of participants in classes II and III). BMI data were unavailable for 7 participants (2.01%). With respect to training habits, almost half of the participants (45.85%) reported a mixed training profile, 25.50% engaged predominantly in strength training, 15.19% in cardio, and 6.02% in stretching-based activity, while 2.01% did not train at all. Most participants trained 1–2 times per week (39.54%) or 3–4 times per week (34.10%); 20.34% trained 5 or more times per week, and 6.02% did not train. Training experience varied widely, ranging from less than 3 months (23.50%) to more than 10 years (12.32%). The mean self-rated training intensity was 6.34 points on a 1–10 scale (*SD* = 1.85; *Me* = 7; *IQR* = 5–7). A cool-down session after training was never performed by 36.68% of participants, performed sometimes by 48.14%, and always by 12.61%. Regarding the training schedule, the single most common pattern was training in the evening (5:00 p.m.–9:00 p.m.; 28.65%), followed by a mixed schedule with a predominance of evening sessions (30.66%). Caffeine intake after 15:00 was reported by more than half of the sample, with 38.11% consuming one portion and 14.04% consuming two or more portions, while 47.85% reported no caffeine intake after 15:00. Of the 349 respondents, 229 (65.62%) were pursuing studies in medical studies, 26 (7.45%) in technical disciplines, 16 (4.58%) in law or economics, 11 (3.15%) in the humanities, 2 (0.57%) in philology, 2 (0.57%) in the natural sciences, and 4 (1.15%) were studying unidentified fields, while 59 (16.91%) were not studying at a university (Table 1).

#### 3.1.1. BDI Results

**Figure 1:**
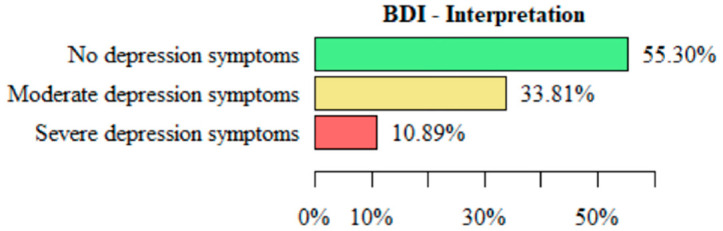
BDI results of study group.

Among the 349 responders included in the study, 193 (55.30%) scored 0–8 points, indicating the absence of depression symptoms; 118 (33.81%) scored 9–28 points, consistent with moderate depression symptoms; and 38 (10.89%) scored 29–63 points, reflecting severe depression symptoms. The study group achieved a mean score of 9.05 points (*SD* = 7.29) (Figure 1).

#### 3.1.2. PSQI Results

**Figure 2:**
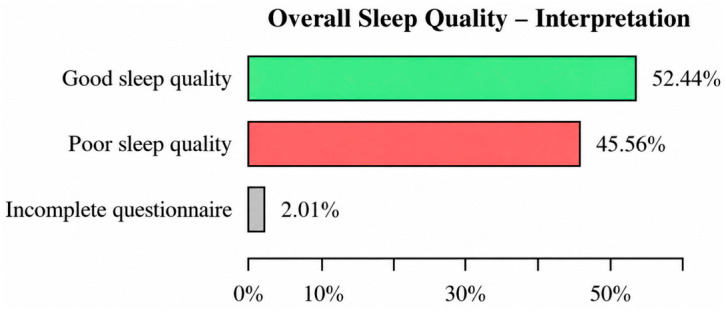
Overall sleep quality results of study group.

The results among the respondents were distributed as follows: of the 349 participants, 183 (52.44%) demonstrated good sleep quality (scores ranged from 0 to 5 points), 159 (45.56%) demonstrated poor sleep quality (scores ranged from 6 to 21 points), and complete data could not be obtained for 7 participants (2.01%). The respondents had a mean score of 5.92 points on the PSQI (*SD* = 3.07) (Figure 2).

**Figure 3:**
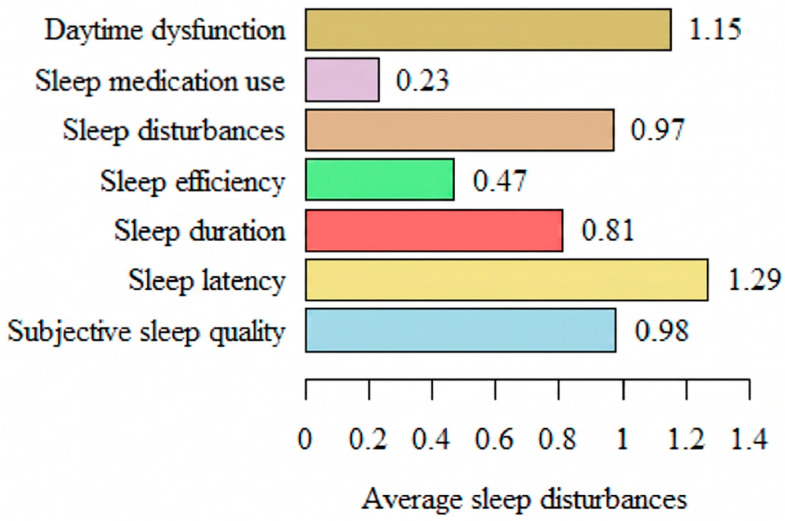
PSQI domains’ results of study group.

The most pronounced difficulties reported by the participants were related to sleep latency (*Me* = 1; *IQR* = 1–2) and daytime dysfunction (*Me* = 1; *IQR* = 1–2), whereas less pronounced difficulties were observed in the domains of subjective sleep quality (*Me* = 1; *IQR* = 1–1), sleep disturbances (*Me* = 1; *IQR* = 1–1), and sleep duration (*Me* = 1; *IQR* = 0–1). The least pronounced difficulties were reported for sleep efficiency (*Me* = 0; *IQR* = 0–1) and use of sleep medication (*Me* = 0; *IQR* = 0–0) (Figure 3).

### 3.2. The Influence of Training Category on PSQI and BDI Scores

The Kruskal–Wallis test showed no statistically significant differences in PSQI scores between the groups engaging in different types of PA across the categories of subjective sleep quality (*p* = 0.964; *p*(*BH*) = 0.968), sleep latency (*p* = 0.235; *p*(*BH*) = 0.627), sleep duration (*p* = 0.199; *p*(*BH*) = 0.627), sleep efficiency (*p* = 0.519; *p*(*BH*) = 0.83), sleep disturbances (*p* = 0.431; *p*(*BH*) = 0.83), use of sleep medication (*p* = 0.968; *p*(*BH*) = 0.968), and overall sleep quality (*p* = 0.735; *p*(*BH*) = 0.968). However, statistically significant differences were observed in the category of daytime dysfunction (*p* = 0.003; *p*(*BH*) = 0.024).

**Figure 4:**
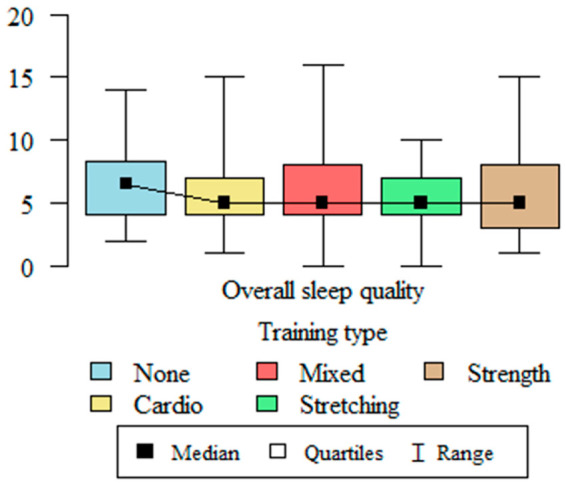
Overall sleep quality results based on training category.

A detailed post hoc analysis (Dunn’s test) revealed that the score in daytime dysfunction category was significantly higher in the non-exercising group (*Me* = 2; *IQR* = 1–2) compared to all other groups: stretching (*Me* = 1; *IQR* = 1–2), mixed training (*Me* = 1; *IQR* = 0.75–2), cardio (*Me* = 1; *IQR* = 1–2), and strength training (*M* = 1; *IQR* = 0–1; *p* < 0.05) (Figure 4).

**Figure 5:**
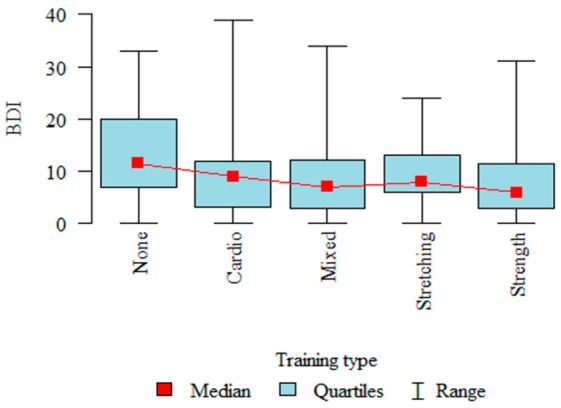
BDI results based on training category.

The Kruskal–Wallis test conducted for the BDI revealed no statistically significant difference (*p* = 0.098) between the groups engaging in different types of PA (Figure 5).

### 3.3. The Effect of Training Frequency on PSQI and BDI Scores

For the purpose of the analysis, four groups were formed based on the number of training sessions per week. The number of training sessions was as follows: 0 for Group A, 1–2 for Group B, 3–4 for Group C, and 5 or more for Group D.

The Kruskal–Wallis test revealed no statistically significant differences in PSQI scores among the analysed groups in the categories of: subjective sleep quality (*p* = 0.204; *p*(*BH*) = 0.408), sleep latency (*p* = 0.351; *p*(*BH*) = 0.562), sleep duration (*p* = 0.686; *p*(*BH*) = 0.784), sleep efficiency (*p* = 0.026; *p*(*BH*) = 0.104), sleep disturbances (*p* = 0.825; *p*(*BH*) = 0.825), use of sleep medication (*p* = 0.563; *p*(*BH*) = 0.751), and overall sleep quality (*p* = 0.039; *p*(*BH*) = 0.104). However, statistically significant differences were observed in the category of daytime dysfunction (*p* = 0.001; *p*(*BH*) = 0.008).

**Figure 6:**
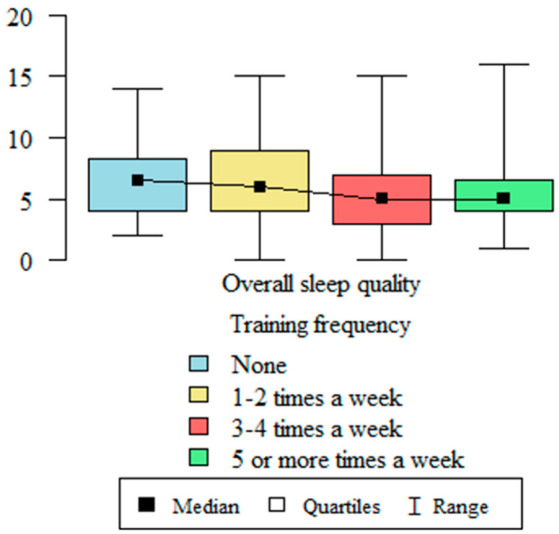
Overall sleep quality results based on training frequency.

A detailed post hoc analysis (Dunn’s test) revealed that the score in the daytime dysfunction category was significantly higher in Group A (*Me* = 2; *IQR* = 1–2) compared to Group B (*Me* = 1; *IQR* = 1–2), Group C (*Me* = 1; *IQR* = 0–2), and Group D (*Me* = 1; *IQR* = 0–1; *p* < 0.05) (Figure 6).

**Figure 7:**
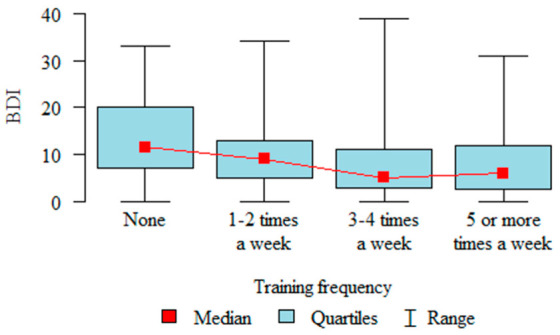
BDI results based on training frequency.

The Kruskal–Wallis test revealed statistically significant differences in BDI scores among the groups (*p* = 0.002). A post hoc analysis (Dunn’s test) indicated that the severity of depressive symptoms was significantly higher in Group A (*Me* = 11.55; *IQR* = 7–20) and Group B (*Me* = 9; *IQR* = 5–13) than in Group C (*Me* = 5; *IQR* = 3–11) and Group D (*Me* = 6; *IQR* = 2.5–11.78; *p* < 0.05) (Figure 7).

### 3.4. The Effect of Training Intensity on PSQI Score

Training intensity was assessed using Spearman’s rank correlation coefficient. The analysis showed a statistically significant negative correlation between training intensity and daytime dysfunction (*r* = −0.220; *p* < 0.001). This association remained statistically significant after Benjamini–Hochberg correction (*p*(*BH*) = 0.008). A weak negative correlation was also observed between training intensity and the global PSQI score (*r* = −0.136; *p* = 0.015); however, this association did not remain statistically significant after correction for multiple comparisons (*p*(*BH*) = 0.060). No statistically significant correlations were found between training intensity and subjective sleep quality (*r* = −0.049; *p* = 0.381), sleep latency (*r* = −0.069; *p* = 0.212), sleep duration (*r* = −0.030; *p* = 0.592), sleep efficiency (*r* = −0.080; *p* = 0.152), sleep disturbances (*r* = −0.005; *p* = 0.932), or sleep medication use (*r* = 0.040; *p* = 0.467) (Table 2).

### 3.5. The Effect of Training Intensity on BDI Score

**Figure 8:**
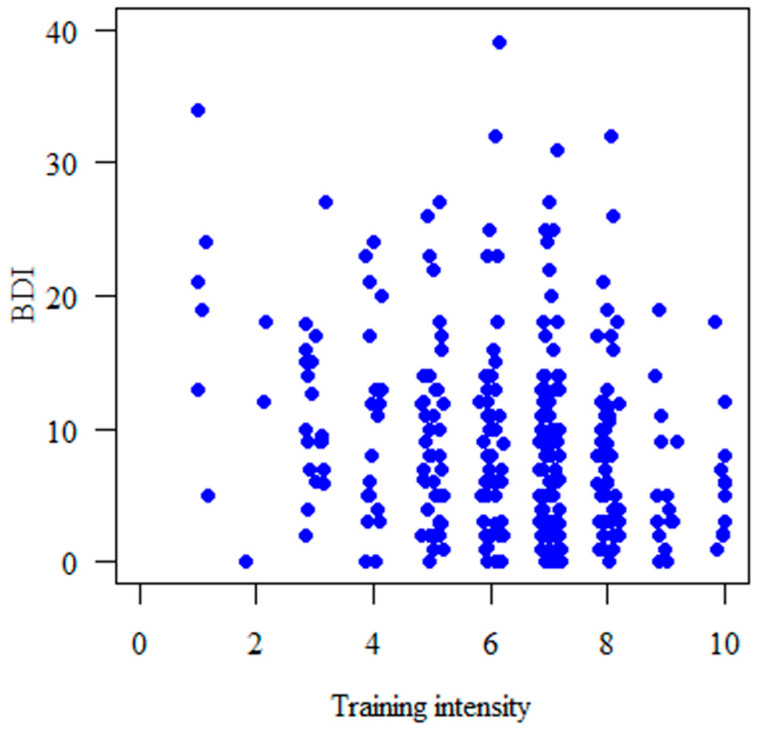
BDI results based on training intensity.

Spearman’s correlation coefficient revealed a statistically significant (*p* < 0.001) association between training intensity and depressive symptoms. Higher training intensity correlated with lower severity of depressive symptoms (*r* = −0.204) (Figure 8).

### 3.6. Correlation Between PSQI Scores and BDI Scores

To evaluate the relationship between sleep disturbances and the severity of depressive symptoms, a correlation analysis was conducted using Spearman’s rank correlation coefficient (*r*). The analysis revealed that the global score and all individual subscales of PSQI correlate positively and statistically significantly with the BDI (*p* < 0.001 for all dimensions). Because higher scores on the PSQI signify greater sleep impairment, these positive correlation coefficients indicate that poorer sleep quality across all domains is consistently associated with an increased severity of depressive symptoms (Table 3).

The strongest association was observed between the overall sleep quality and depressive symptoms (*r* = 0.574, *p* < 0.001), demonstrating a robust relationship where overall worse sleep quality strongly tracks with higher depression severity. Notable moderate-to-strong positive correlations were also found for daytime dysfunction (*r* = 0.489, *p* < 0.001) and subjective sleep quality (*r* = 0.441, *p* < 0.001), suggesting that impairments in daily functioning due to sleepiness and a poor subjective perception of sleep are particularly prominent markers of depressive symptom severity.

Furthermore, statistically significant positive correlations were established for the remaining PSQI subscales. These include sleep latency (*r* = 0.349, *p* < 0.001), sleep disturbances (*r* = 0.259, *p* < 0.001), sleep duration (*r* = 0.241, *p* < 0.001), sleep medication use (*r* = 0.196, *p* < 0.001), and sleep efficiency (*r* = 0.180, *p* = 0.001). Although these relationships are weaker to moderate in magnitude, they remain highly significant, indicating that prolonged sleep onset, frequent nocturnal awakenings, shorter sleep duration, reliance on pharmacotherapy, and lower sleep efficiency are all significantly linked to higher levels of depressive symptoms.

### 3.7. Correlation Between the Cool-Down Session and PSQI Scores

**Figure 9:**
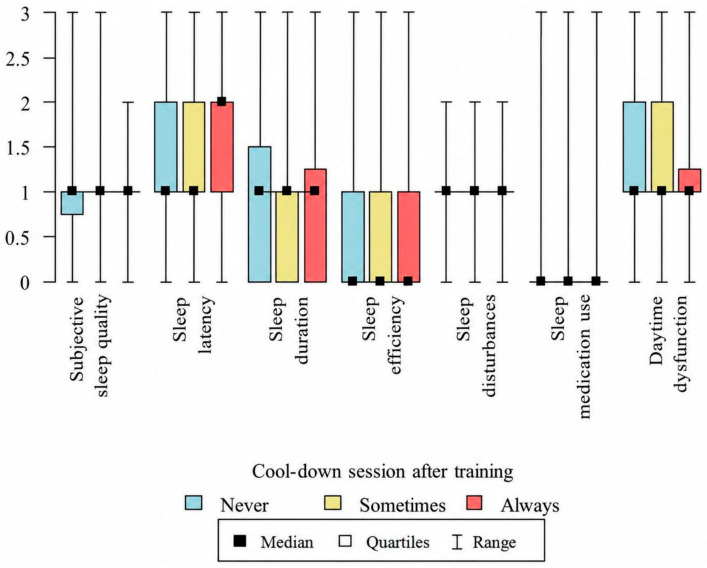
Correlation between cool-down sessions and PSQI domains scores.

**Figure 10:**
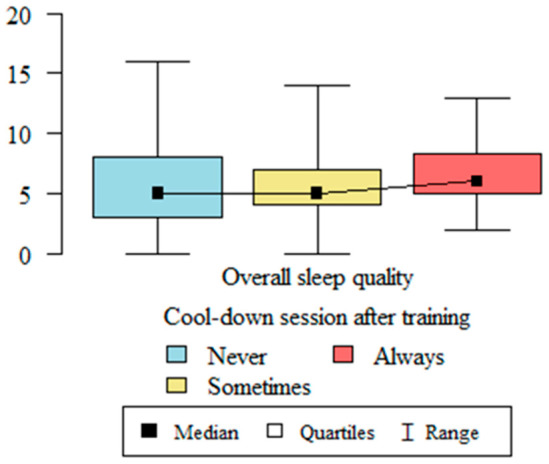
Correlation between cool-down sessions and overall sleep quality.

For the purposes of the analysis, three groups were created. The aforementioned groups consisted of individuals who indicated in the survey that they never use cool-down sessions (A), those who do so sometimes (B), and those who always do so (C). A Kruskal–Wallis test with Dunn’s post hoc analysis was conducted. The score on the sleep latency domain of the PSQI questionnaire was significantly higher (*p* = 0.026) among those who always used cool-down sessions (*Me* = 2; *IQR* = 1–2) than among those who used them sometimes (*Me* = 1; *IQR* = 1–2) or never (*Me* = 1; *IQR* = 1–2). The results for the remaining domains were not statistically significant (Figure 9 and Figure 10).

### 3.8. Correlation Between Training Schedule and PSQI

Regarding the impact of the training schedule, groups A-F were established based on the hours of training sessions. Group A corresponded to morning training (05:00 a.m.–10:00 a.m.), Group B to afternoon training (10:00 a.m.–5:00 p.m.), Group C to evening training (5:00 p.m.–9:00 p.m.), and Group D to night training (after 9:00 p.m.). Group E comprised mixed schedules with a predominance of morning sessions, while Group F included mixed schedules with a predominance of evening sessions.

**Figure 11:**
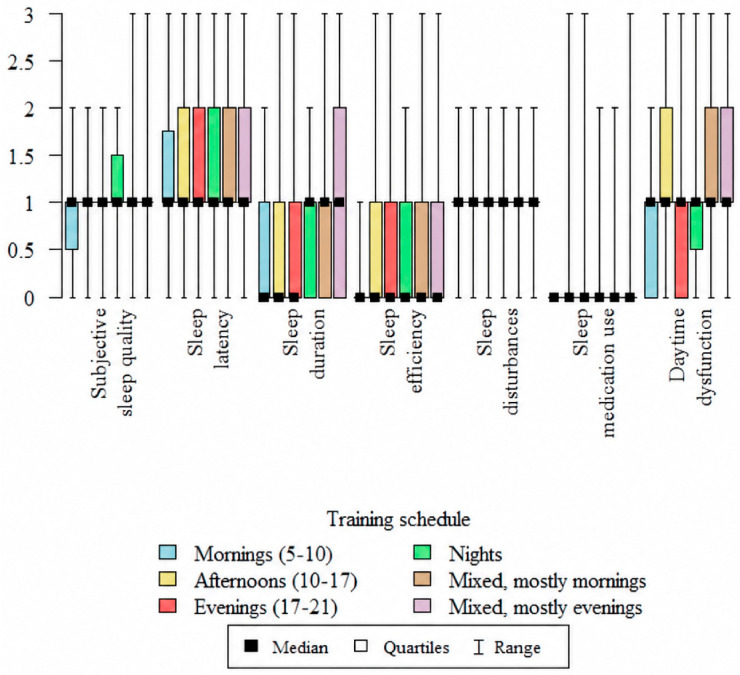
Correlation between training schedule and PSQI domains scores.

**Figure 12:**
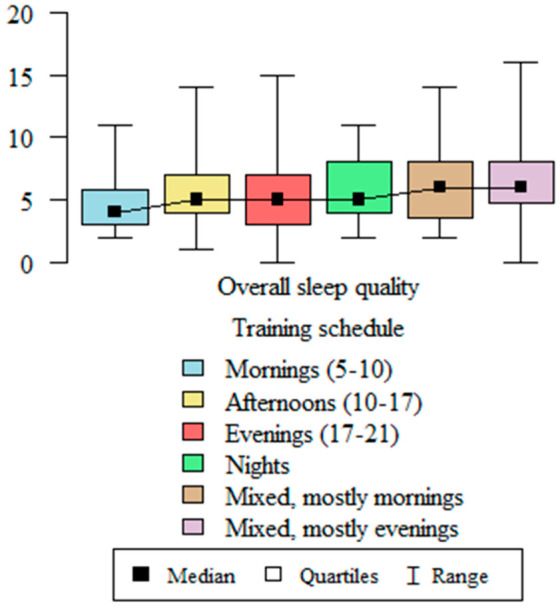
Correlation between training schedule and overall sleep quality.

A Kruskal–Wallis test with Dunn’s post hoc analysis was conducted. The score for the daytime dysfunction domain of the PSQI questionnaire was significantly higher (*p* = 0.016) among individuals training at mixed hours with a predominance of evening sessions (*Me* = 1; *IQR* = 1–2) than among those training in the afternoon (*Me* = 1; *IQR* = 1–2) or evening (*Me* = 1; *IQR* = 0–1). The results for the remaining domains were not statistically significant (Figure 11 and Figure 12).

### 3.9. Correlation Between Caffeine Consumption and PSQI

To assess the impact of caffeine consumption after 3:00 p.m. on sleep, respondents were divided into three groups based on their self-reported caffeine intake. Group A consisted of individuals who did not consume caffeine after 3:00 p.m., Group B included those who consumed one serving (80 mg) of caffeine, and Group C comprised those who consumed two or more servings.

**Figure 13:**
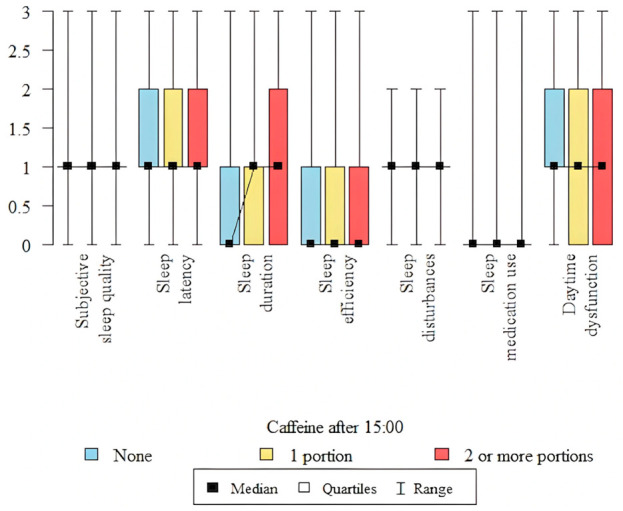
Correlation between caffeine consumption and PSQI domains scores.

**Figure 14:**
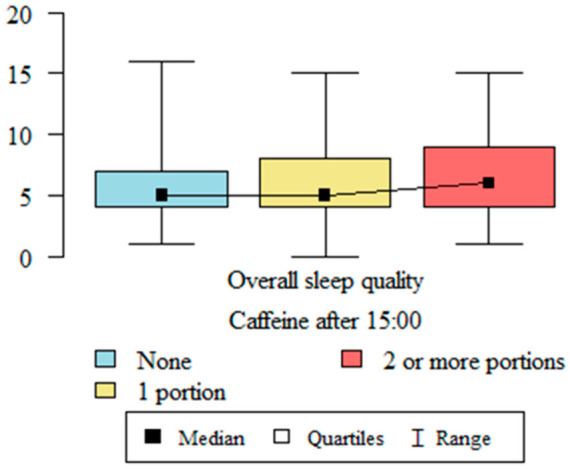
Correlation between caffeine consumption and overall sleep quality.

A Kruskal–Wallis test with Dunn’s post hoc analysis was conducted. Scores on the sleep duration domain of the PSQI questionnaire were significantly higher (*p* = 0.004) among those consuming two or more servings of caffeine after 3:00 p.m. (*Me* = 1; *IQR* = 0–2), as well as among those consuming one serving (*Me* = 1; *IQR* = 0–1), compared to individuals who did not consume caffeine after 3:00 p.m. (*Me* = 0; *IQR* = 0–1). The results for the remaining domains were not statistically significant (Figure 13 and Figure 14).

### 3.10. Correlation Between the Length of Training Experience and Depression

**Figure 15:**
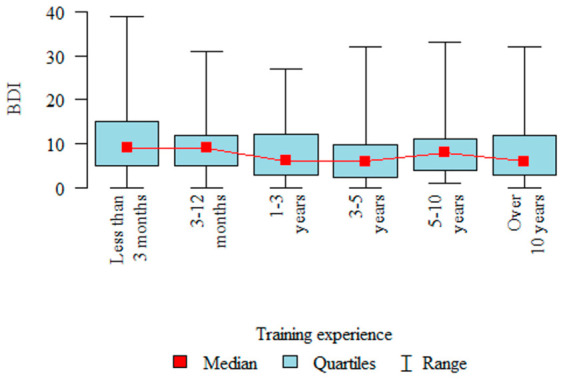
Correlation between the length of training experience and BDI scores.

For the analysis of training experience, six groups were created. Group A included participants training for less than 3 months, group B included those with 3–12 months of training experience, group C included those with 1–3 years of training experience, group D included those with 3–5 years of training experience, group E included those with 5–10 years of training experience, and group F included participants training for more than 10 years. The Kruskal–Wallis test showed statistically significant differences in BDI scores between the analysed groups (*p* = 0.048). Dunn’s post hoc test showed significantly higher BDI scores in group A (*Me* = 9.00; *IQR* = 5.00–15.00) than in group D (*Me* = 6.00; *IQR* = 2.25–9.75), and in group B (*Me* = 9.00; *IQR* = 5.00–12.00) than in group D (*Me* = 6.00; *IQR* = 2.25–9.75) (both *p* < 0.05). Median BDI scores in the remaining groups were as follows: group C (*Me* = 6.15; *IQR* = 3.00–12.25), group E (*Me* = 8.00; *IQR* = 4.00–11.00), and group F (*Me* = 6.00; *IQR* = 3.00–12.00). These findings indicate that significantly higher BDI scores were observed in participants with less than 12 months of training experience than in those with 3–5 years of training experience (Figure 15).

### 3.11. Physical Activity and Its Direct and Indirect Impact on Depression

**Figure 16:**
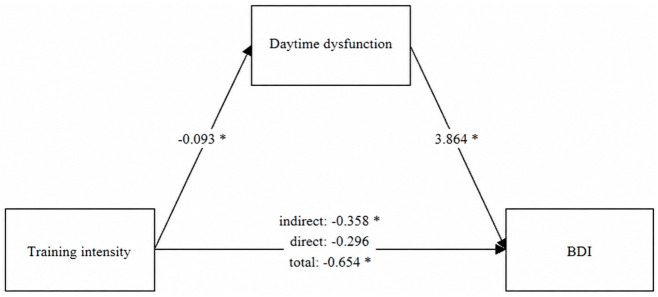
Mediation model of the impact of training intensity on BDI through daytime dysfunction. * statistically significant.

To assess the relationship between PA intensity and BDI scores, a series of mediation analyses was performed using structural equation modelling. The analysis included 328 participants. Each of the seven PSQI domains and the global PSQI score (overall sleep quality) was tested separately as the mediating variable (M), with training intensity as the independent variable (X), and the BDI score as the dependent variable (Y). Moreover, the tests were performed on both unadjusted and adjusted models. Higher training intensity was significantly (*p*(*BH*) = 0.01) associated with reduced depressive symptoms indirectly by lowering daytime dysfunction (−0.358) in the adjusted model. The overall sleep quality score seemed to be significant in the adjusted model (*p* = 0.044); however, the Benjamini–Hochberg test proved it insignificant (*p*(*BH*) = 0.176). Other indirect effects were statistically insignificant (*p* > 0.05), both in unadjusted and adjusted models (Figure 16; Table 4).

## 4. Discussion

The findings of the present study support the widely discussed role of PA as an important correlate of mental health, demonstrating a distinct statistical association with lower levels of depressive symptoms. Analysis of training experience highlights that this relationship may be more pronounced over time; specifically, participants with an established training history of 3–5 years reported lower symptom severity compared to cohorts with shorter athletic experience of less than a year (Figure 15). This suggests that the physiological and psychological benefits of exercise accumulate gradually, requiring sustained regular practice to be fully reflected in self-reported well-being. Furthermore, looking at the volume of exercise, a clear pattern emerged regarding training frequency: individuals who do not engage in PA or train only 1–2 times a week exhibited significantly higher BDI scores compared to those exercising 3 or more times per week. These findings resonate with the meta-analysis published by A. Heissel et al., which demonstrated that PA is strongly tied to a reduction in depressive symptoms, with its relevance largely depending on the appropriate selection of exercise intensity and regularity [19].

Although the central hypothesis of this study presumed that PA intensity is associated with lower depressive symptoms specifically through the pathway of improved sleep quality, the present data did not support such an indirect or mediating mechanism. When adjusting for multiple comparisons, the exploratory path coefficients between training intensity and the majority of nocturnal sleep metrics, such as sleep latency, duration, and efficiency, did not retain statistical significance (*p*(*BH*) > 0.05). This contrasts with longitudinal frameworks, such as the model described by K. Kaseva et al. [20], which position sleep disturbances as the primary link between physical inactivity and low mood. Instead, within this sample, the inverse relationship between subjectively rated training intensity and depressive symptoms (*r* = −0.204, *p* < 0.001) operates independently of changes in sleep architecture [20]. This standalone association aligns with the documented biological correlates of movement described in the literature, which include the stimulation of brain neuroplasticity, the regulation of neurotransmitters, and the reduction in inflammatory marker levels [21].

Because these variables were measured concurrently, this link must be interpreted strictly as a cross-sectional co-occurrence, meaning that while intense exercise tracks with lower symptom scores, it is equally plausible that individuals experiencing minimal depressive symptoms simply possess a greater capacity to maintain demanding exercise routines.

Concurrently, a separate and highly pronounced relationship was observed between subjectively reported sleep disturbances and the severity of depressive symptoms. Our analyses revealed that every single dimension of sleep disturbance measured by the PSQI is significantly and positively associated with an increase in the depression index on the BDI scale (Table 3). This strong co-occurrence mirrors the findings of K. Kaseva et al., who demonstrated that nocturnal rest problems are inextricably and bidirectionally linked to low mood [20]. This robust comorbidity is further substantiated by data from clinical outpatient populations, where clinically significant sleep impairments are highly prevalent and reach their peak specifically among individuals diagnosed with depressive disorders [6]. Interestingly, the global PSQI score demonstrated the strongest statistical association with depressive symptoms, followed closely by daytime dysfunction and subjective sleep quality. This pattern strongly implies that the subjective perception of sleep deprivation and its immediate toll on daily responsibilities share a much tighter cross-sectional link with depressive symptoms than purely quantitative metrics, given that sleep duration (*r* = 0.241) and sleep efficiency (*r* = 0.180) demonstrated noticeably weaker associations.

The subjectively perceived higher training intensity was associated with less severe depressive symptoms and lower daytime dysfunction scores. The correlation between training intensity and the PSQI global score was not significant after the Benjamini–Hochberg correction. Our results are partially consistent with another publication on this topic, whose authors emphasise the key value of training intensity for both sleep quality and depressive symptoms in the population of students diagnosed with anxiety disorders [22]. Similar results were reported in a study conducted among adolescents [23]. Despite the demographic differences, higher intensity training was found to be correlated with better sleep quality. The discrepancy between our findings and the existing, albeit scarce, literature may stem from differences in how training intensity was measured. While both of the cited studies utilised objective measures, with the one conducted among adolescents additionally employing a standardised questionnaire for self-rated intensity, our study relied on a 1–10 scale.

Numerous reports in the literature confirm the connection between PA and sleep. In a recent study conducted on a large group of students, Zhang et al. demonstrated that PA contributes to enhanced self-esteem and better emotional control, which indirectly improves sleep quality [24]. Similar results were obtained in another study, where Chang et al. showed a beneficial effect of PA on sleep and a reduction in depressive symptoms [25]. A systematic review by Alnawwar et al. [26], which ultimately included 23 publications, proved that regular PA positively affects sleep quality and shortens the time it takes to fall asleep. The authors also point out that moderate-intensity exercise yields the best results, as high-intensity workouts can lead to difficulties falling asleep, especially when performed shortly before bedtime. Consequently, scheduling moderate-intensity PA in the morning or afternoon might be considered the best approach. Additionally, a tendency toward sleep onset difficulties was observed when daily PA exceeded 90 min [26].

Furthermore, the relationship observed in our study regarding training experience, specifically, that lower BDI scores were identified in participants with 3–5 years of experience compared to those with less than 12 months, finds general conceptual support in the literature. For instance, a comprehensive review by Budde et al. [27] analysed the biological mechanisms underlying the impact of sports on the brain, noting that regular PA co-occurs with restorative processes in the nervous system, including neuroplasticity and the modulation of stress hormones. Their work suggests that such structural changes typically require extended periods of continuous stimulation [27]. While our cross-sectional data cannot establish a definitive temporal timeline or prove that a specific duration is required to trigger biological protection, the lower symptom reports observed specifically within the 3–5 year cohort suggest that established, multi-year exercise routines share a more robust statistical link with lower depressive symptoms than short-term practice. This underscores the importance of considering long-term habituation and adherence, rather than focusing solely on immediate training characteristics, when examining PA in relation to mental well-being.

Our findings show that higher training frequency may be another factor that is associated with lower depressive symptoms. Respondents who did not exercise or train less than three times a week had significantly higher scores on the BDI. A research team from Korea achieved similar results. In their study, the authors used the Korean version of the CES-D scale to obtain results indicating that the population exercising three or more times a week had fewer depressive symptoms than the non-exercising group or the group exercising 1–2 times a week [28]. After applying the Benjamini–Hochberg correction for multiple comparisons, daytime dysfunction was the only PSQI domain that remained statistically significant; the scores were significantly higher in the non-training group compared to the training groups. A study conducted on a population of college students diagnosed with anxiety disorder found that training frequency had no effect on the PSQI but correlated with lower depressive symptoms [22]. Contrary to our findings, Santos et al. [29] presented results indicating that the non-training group exhibited a higher global PSQI score compared to the training groups. Their study cohort was divided into three categories: non-training, training 1–3 times a week, and training 4–7 times a week. While significant differences in specific PSQI domains were observed between the training and non-training groups, no such differences were found between the two training groups (1–3 vs. 4–7 times per week) [29].

In the present study, no statistically significant differences were observed between the self-reported PA profile groups in BDI scores or the global PSQI score. Likewise, no statistically significant differences were found for six of the seven PSQI domains. The only exception was daytime dysfunction, for which participants reporting no PA obtained significantly higher scores than those in the cardio, mixed, stretching, and strength-training groups (*p* = 0.003) (Figure 4 and Figure 5).

Our results regarding the BDI differ from those reported in the network meta-analysis by Noetel et al. [11], in which the type of exercise influenced the magnitude of the antidepressant effect in participants. The authors demonstrated the greatest reduction in depressive symptoms compared to active control groups among those practising walking or running, yoga, or strength training [11]. In the context of our data, walking or running most closely corresponds to the cardio group, whereas strength training is most closely comparable to the strength group used in our classification. Based on this, one might have expected individuals in these groups to achieve lower BDI scores than non-exercisers or participants engaging in less clearly comparable forms of PA. However, no such relationship was found in our study. A possible explanation is that Noetel et al. [11] demonstrated the advantage of specific forms of PA, particularly when performed at a higher intensity. Therefore, the lack of significant differences between the groups categorised by the dominant type of activity in our study should not be interpreted as evidence that training parameters are irrelevant. It should be emphasised that PA intensity was analysed separately in our study, and its relationship with BDI scores is discussed in a separate section of the manuscript. In this section, however, the self-reported activity profile was assessed, which may be a less precise indicator when analysed as a separate parameter.

Regarding the PSQI, our results partially align with the network meta-analysis by Wang et al. [30], which found no statistically significant differences in efficacy among some of the analysed exercise modalities, despite their beneficial effects on sleep quality compared to control groups. Similarly, in our study, the self-reported PA profile alone did not significantly differentiate the global PSQI score or scores in six of the seven PSQI domains [30]. The only exception was daytime dysfunction, for which non-exercising participants had significantly higher scores than participants in the cardio, mixed, stretching, and strength groups. One possible explanation is that daytime dysfunction may be more closely related to engagement in regular PA than to the specific type of exercise performed. Consequently, differences may be more apparent between physically active and inactive individuals than between specific exercise modalities. However, other meta-analyses have reported different findings. Nevertheless, based on previous evidence, the cardio and strength groups were expected to represent the exercise modalities most likely to demonstrate favourable sleep outcomes. Gao et al., in a network meta-analysis involving middle-aged and older adults, identified aerobic exercise as the most effective modality for improving the global PSQI score [31]. In contrast, Bahalayothin et al., in a study focusing on older adults with insomnia, demonstrated that the greatest reduction in PSQI was achieved with strengthening exercises, followed by aerobic and combined exercise interventions [32]. However, no such differences were observed in the present study. One possible explanation for this discrepancy is that classification according to the dominant self-reported activity profile did not account for differences in training intensity, frequency, duration, weekly training volume, or adherence, all of which may have had a greater influence on sleep-related outcomes than exercise modality itself. In addition, the activity categories used in the present study were relatively broad and could encompass substantially different exercise patterns. For example, the cardio group could include both recreational walking and vigorous running, despite the markedly different physiological demands of these activities. Therefore, the lack of significant differences between groups should not be interpreted as evidence that all forms of PA are similarly associated with BDI and PSQI outcomes. Because these studies were conducted in different populations and used interventional designs, their findings should be considered only as a reference point and not as direct comparators for the present cohort of young adults.

A crucial aspect of physical training involves concluding exercise with a cool-down session. Post-exercise cooling down significantly affects the ease of sleep onset among the surveyed participants. Importantly, the frequency of these cool-down sessions is reflected in the outcomes; specifically, respondents who incorporated cool-down practices only after certain training sessions achieved lower scores in the sleep latency domain of the PSQI compared to those who cooled down after every workout. However, their scores were still significantly higher than those of the group that did not engage in cool-down sessions at all. A narrative review by Van Hooren et al. questions the efficacy of post-exercise cool-down sessions, suggesting they exert negligible effects on physiological recovery and sleep-related parameters [33]. Concurrently, individuals who incorporated cool-down sessions into their training regimens may have accumulated greater total exercise volume and intensity compared to those who did not engage in active recovery. This finding is particularly salient when viewed through the prism of our study’s target population, wherein 63.61% of respondents reported training after 5:00 p.m. or predominantly during evening hours (Table 1). The phenomenon under analysis is relatively underreported in the current literature, as most available studies focus solely on training intensity; therefore, further research in this domain is warranted.

The prominent link with daytime dysfunction requires a cautious interpretation. This specific PSQI domain conceptually overlaps with core diagnostic criteria for depression, such as a lack of energy, decreased motivation, and concentration difficulties [34]. This shared variance may partially inflate the statistical association observed on the BDI scale (Table 3). Nevertheless, the differences identified across training schedules point to a compelling interaction. Participants with mixed, mostly evening routines reported significantly higher daytime dysfunction than those training in the afternoons or regular evenings. This pattern supports the hypothesis that PA can act as an effective external circadian time cue, or zeitgeber [35]. Since circadian dysregulation is a well-established component in the pathophysiology of depression, appropriately timed exercise may closely co-occur with better daytime alertness [22]. However, higher depressive scores were also linked to multifaceted disruptions across the entire sleep structure, including prolonged sleep latency and frequent nighttime awakenings. This underscores that within this young adult convenience sample, sleep disturbances reflect a comprehensive degradation of rest rather than an isolated nocturnal complaint.

The time of day at which training occurs may also play a role in overall well-being (Figure 12). This outcome may be attributed to the structure of the study population, which consisted largely of medical students. These individuals frequently exhibit irregular daily schedules due to the nature of their university courses and part-time employment. Consequently, they may face difficulties in maintaining a consistent workout schedule. Conversely, individuals who train regularly at the same time achieve lower scores in this domain. This finding aligns with existing literature regarding the impact on chronobiology, given that disruptions in these patterns can lead to poorer daytime functioning [35,36,37]. Interestingly, in our study, no statistically significant effect of training exclusively during night and evening hours was observed on the remaining PSQI domains. This is consistent with the findings of current research, which lacks a definitive consensus regarding the impact of evening workouts on sleep [38,39].

Respondents who consumed caffeine after 3:00 p.m. exhibited a shorter sleep duration. This effect escalated with an increase in the amount of caffeine ingested. Somewhat surprisingly, no significant impact of caffeine was demonstrated on the remaining domains of the PSQI. This can be interpreted in two ways: either a late timing of caffeine intake reduces sleep duration, or individuals with shorter sleep duration more frequently consume caffeine during later hours to maintain attention and focus in the afternoon and evening. However, the latter hypothesis is not reflected in the remaining PSQI outcomes; no statistically significant association was found between caffeine doses after 3:00 p.m. and daytime functioning. While caffeine consumption itself has a well-documented negative impact on sleep quality, many individuals remain unaware of its clearance half-life [40]. Since caffeine is a common ingredient in pre-workout formulas and dietary supplements used by physically active individuals, it can be hypothesised that some subjects inadvertently ingested even higher doses than those declared in the survey. Singla B. et al. [41] conducted a study on a similar cohort of medical students, evaluating the impact of caffeine consumption at various times on PSQI scores. In their study, the group consuming caffeine later in the day had significantly higher PSQI scores compared to the early-consumption group; however, the exact milligram content per serving was not specified, and the PSQI scores were not disaggregated into individual subdomains [41].

## 5. Limitations

Despite the numerous strengths of this study, several limitations must be acknowledged. First, the study design, which relied on convenience sampling via the distribution of a survey link across the university campus and recruitment among acquaintances, introduces a potential selection bias. This resulted in a highly homogeneous study population consisting predominantly of young adults, the majority of whom were medical students at the Poznań University of Medical Sciences. Furthermore, the survey was primarily targeted at physically active individuals, as the core objective was to determine optimal training parameters. Consequently, this led to an underrepresentation of non-exercising individuals, which reduced the statistical power of comparisons between the non-exercising group and the training cohorts. Additionally, while the anonymous nature of the online survey encouraged unconstrained responses, it is worth noting that some respondents may have completed the questionnaire perfunctorily, potentially compromising data reliability.

A further limitation of this study is its cross-sectional design. Because exposure, mediator and outcome were all assessed at a single time point and largely by self-report, the study is reported as an association study, and the temporal ordering of the observed relationships cannot be established from these data. Consequently, because the data are cross-sectional, these models are presented as exploratory path analyses and do not establish temporal or causal mediation. Therefore, reverse causation cannot be excluded, and it is equally plausible that poorer sleep quality or greater depressive symptom severity may influence PA behaviours.

Regarding the methodology, a custom-designed questionnaire was utilised to assess training parameters, as existing validated scales did not meet the specific objectives of this study. Habitual retrospective average training intensity was assessed using a 10-point scale. The retrospective nature of this scale may result in recall bias. Incorporating more quantifiable metrics of exercise intensity could enhance the validity of the findings. Utilising a pre-validated questionnaire could mitigate the risk of potential linguistic ambiguities within the items. Insights gained during the data analysis stage suggest that future instruments would benefit from the inclusion of precise operational definitions (e.g., clarifying how respondents conceptualise and execute cool-down sessions) as well as a more rigorous categorisation of specific sport disciplines. Such refinements would better facilitate the integration and comparison of the collected data with existing literature in the field.

A methodological limitation is that the ordinal PSQI component scores (0–3) were modelled as continuous in the path analyses. While this is common practice and parametric methods are robust to ordinal inputs of this kind, it remains a simplification. Reassuringly, the key associations were consistent between the non-parametric analyses (Spearman correlations, Kruskal–Wallis tests) and the path models [18].

A significant limitation of this study is the potential for residual confounding. Due to the utilisation of convenience sampling, the number of questionnaire items was intentionally limited to minimise respondent burden and mitigate non-response bias. Consequently, certain aspects warrant further refinement; specifically, items covered by open-ended questions could have been structured as closed-ended checklists. Furthermore, participants were not requested to specify their psychiatric conditions or history of psychiatric treatment, as such inquiries were deemed potentially stigmatising. Because these unmeasured factors are intrinsically linked to both depression and sleep disorders, and were not fully controlled for in our statistical models, the observed associations may be subject to residual confounding.

## 6. Conclusions

Our research focused mainly on identifying specific features of training that could be associated with better sleep quality and less severe depressive symptoms. This exploratory study provides interesting results regarding the association between depressive symptoms, training intensity and frequency. Higher training intensity and frequency were associated with reduced depressive symptoms. Moreover, our results did not support sleep as a mediator in the relationship between physical activity and depressive symptoms. Results regarding training experience point to an important detail, that physical training was associated with less severe depressive symptoms only after an extended period of time. Our study creates a framework for future research. Future longitudinal studies should investigate specific training regimens to determine which parameters are most effective in maximising these mental health outcomes. Our findings align with the growing public and clinical interest in lifestyle medicine and highlight the interconnectedness of physical and mental health.

## Figures and Tables

**Table 1:** Group sample.

Parameter		Total (N = 349)
Sex	Female	215 (61.60%)
	Male	133 (38.11%)
	Unknown	1 (0.29%)
Age [years]	Mean (SD)	23.06 (6.5)
	Median (quartiles)	21 (20–23)
	Range	16–54
	n	345
BMI [kg/m^2^]	Mean (SD)	22.44 (3.56)
	Median (quartiles)	22.15 (19.95–24.24)
	Range	15.74–40.14
	n	342
BMI	Thinness	8 (2.29%)
	Underweight	26 (7.45%)
	Normal weight	245 (70.20%)
	Overweight	48 (13.75%)
	Obesity	13 (3.72%)
	Obesity, class II	1 (0.29%)
	Obesity, class III	1 (0.29%)
	Unknown	7 (2.01%)
Caffeine after 3:00 p.m.	None	167 (47.85%)
	1 portion	133 (38.11%)
	2 or more portions	49 (14.04%)
Training type	None	21 (6.02%)
	Cardio	53 (15.19%)
	Mixed	160 (45.85%)
	Stretching	21 (6.02%)
	Strength	89 (25.50%)
	Unknown	5 (1.43%)
Training frequency	None	21 (6.02%)
	1–2 times a week	138 (39.54%)
	3–4 times a week	119 (34.10%)
	5 or more times a week	71 (20.34%)
Training experience	Less than 3 months	82 (23.50%)
	3–12 months	61 (17.48%)
	1–3 years	68 (19.48%)
	3–5 years	58 (16.62%)
	5–10 years	29 (8.31%)
	Over 10 years	43 (12.32%)
	Unknown	8 (2.29%)
Cool-down session after training	Never	128 (36.68%)
	Sometimes	168 (48.14%)
	Always	44 (12.61%)
	Unknown	9 (2.58%)
Training intensity	Mean (SD)	6.34 (1.85)
	Median (quartiles)	7 (5–7)
	Range	1–10
	n	328
Training schedule	Mornings (5:00 a.m.–10:00 a.m.)	11 (3.15%)
	Afternoons (10:00 a.m.–5:00 p.m.)	69 (19.77%)
	Evenings (5:00 p.m.–9:00 p.m.)	100 (28.65%)
	Nights (after 9:00 p.m.)	15 (4.30%)
	Mixed, mostly mornings	39 (11.17%)
	Mixed, mostly evenings	107 (30.66%)
	Unknown	8 (2.29%)

**Table 2:** Effects of training intensity on PSQI component scores.

PSQI	Training Intensity
	Spearman’s Correlation Coefficient
Subjective sleep quality	*r* = −0.049, *p* = 0.381, *p*(*BH*) = 0.61
Sleep latency	*r* = −0.069, *p* = 0.212, *p*(*BH*) = 0.424
Sleep duration	*r* = −0.03, *p* = 0.592, *p*(*BH*) = 0.677
Sleep efficiency	*r* = −0.08, *p* = 0.152, *p*(*BH*) = 0.405
Sleep disturbances	*r* = −0.005, *p* = 0.932, *p*(*BH*) = 0.932
Sleep medication use	*r* = 0.04, *p* = 0.467, *p*(*BH*) = 0.623
Daytime dysfunction	*r* = −0.22, *p* < 0.001 *, *p*(*BH*) = 0.008 *
Overall sleep quality	*r* = −0.136, *p* = 0.015 *, *p*(*BH*) = 0.06

* statistically significant (*p* < 0.05).

**Table 3:** Correlation between PSQI domains scores and BDI score.

PSQI	BDI
	Spearman’s Correlation Coefficient
Subjective sleep quality	*r* = 0.441, *p* < 0.001 *
Falling asleep	*r* = 0.349, *p* < 0.001 *
Sleep duration	*r* = 0.241, *p* < 0.001 *
Sleep efficiency	*r* = 0.18, *p* = 0.001 *
Sleep disturbances	*r* = 0.259, *p* < 0.001 *
Sleep medication use	*r* = 0.196, *p* < 0.001 *
Daytime functioning disorder	*r* = 0.489, *p* < 0.001 *
Overall sleep quality	*r* = 0.574, *p* < 0.001 *

* statistically significant (*p* < 0.05).

**Table 4:** Parameters of mediation models for the relationship between training intensity and BDI scores with PSQI domains as mediators.

Dependent Variable (Y)	Independent Variable (X)	Mediator (M)	Unadjusted Model			Adjusted Model		
			Direct Effect	Indirect Effect	Total Effect	Direct Effect	Indirect Effect	Total Effect
BDI	Training intensity	Subjective sleep quality	−0.756 (−1.117; −0.395) *p* < 0.001 * *p*(*BH*) < 0.001 *	−0.126 (−0.31; 0.058) *p* = 0.178 *p*(*BH*) = 0.238	−0.882 (−1.286; −0.479) *p* < 0.001 * *p*(*BH*) < 0.001 *	−0.547 (−0.934; −0.16) *p* = 0.006 * *p*(*BH*) = 0.008 *	−0.108 (−0.317; 0.102) *p* = 0.314 *p*(*BH*) = 0.527	−0.654 (−1.093; −0.215) *p* = 0.003 * *p*(*BH*) = 0.003 *
BDI	Training intensity	Sleep latency	−0.761 (−1.136; −0.386) *p* < 0.001 * *p*(*BH*) < 0.001 *	−0.122 (−0.279; 0.036) *p* = 0.13 *p*(*BH*) = 0.214	−0.882 (−1.286; −0.479) *p* < 0.001 * *p*(*BH*) < 0.001 *	−0.572 (−0.981; −0.163) *p* = 0.006 * *p*(*BH*) = 0.008 *	−0.083 (−0.248; 0.083) *p* = 0.329 *p*(*BH*) = 0.527	−0.654 (−1.093; −0.215) *p* = 0.003 * *p*(*BH*) = 0.003 *
BDI	Training intensity	Sleep duration	−0.794 (−1.185; −0.404) *p* < 0.001 * *p*(*BH*) < 0.001 *	−0.088 (−0.201; 0.025) *p* = 0.128 *p*(*BH*) = 0.214	−0.882 (−1.286; −0.479) *p* < 0.001 * *p*(*BH*) < 0.001 *	−0.618 (−1.042; −0.195) *p* = 0.004 * *p*(*BH*) = 0.008 *	−0.036 (−0.153; 0.081) *p* = 0.548 *p*(*BH*) = 0.731	−0.654 (−1.093; −0.215) *p* = 0.003 * *p*(*BH*) = 0.003 *
BDI	Training intensity	Habitual sleep efficiency	−0.817 (−1.215; −0.419) *p* < 0.001 * *p*(*BH*) < 0.001 *	−0.065 (−0.15; 0.02) *p* = 0.134 *p*(*BH*) = 0.214	−0.882 (−1.286; −0.479) *p* < 0.001 * *p*(*BH*) < 0.001 *	−0.61 (−1.042; −0.177) *p* = 0.006 * *p*(*BH*) = 0.008 *	−0.045 (−0.13; 0.04) *p* = 0.299 *p*(*BH*) = 0.527	−0.654 (−1.093; −0.215) *p* = 0.003 * *p*(*BH*) = 0.003 *
BDI	Training intensity	Sleep disturbances	−0.876 (−1.265; −0.487) *p* < 0.001 * *p*(*BH*) < 0.001 *	−0.006 (−0.114; 0.101) *p* = 0.911 *p*(*BH*) = 0.911	−0.882 (−1.286; −0.479) *p* < 0.001 * *p*(*BH*) < 0.001 *	−0.67 (−1.093; −0.248) *p* = 0.002 * *p*(*BH*) = 0.008 *	0.016 (−0.104; 0.137) *p* = 0.794 *p*(*BH*) = 0.907	−0.654 (−1.093; −0.215) *p* = 0.003 * *p*(*BH*) = 0.003 *
BDI	Training intensity	Use of sleeping medication	−0.906 (−1.302; −0.509) *p* < 0.001 * *p*(*BH*) < 0.001 *	0.023 (−0.052; 0.099) *p* = 0.543 *p*(*BH*) = 0.621	−0.882 (−1.286; −0.479) *p* < 0.001 * *p*(*BH*) < 0.001 *	−0.653 (−1.082; −0.224) *p* = 0.003 * *p*(*BH*) = 0.008 *	−0.001 (−0.094; 0.091) *p* = 0.978 *p*(*BH*) = 0.978	−0.654 (−1.093; −0.215) *p* = 0.003 * *p*(*BH*) = 0.003 *
BDI	Training intensity	Daytime dysfunction	−0.441 (−0.81; −0.072) *p* = 0.019 * *p*(*BH*) = 0.019 *	−0.441 (−0.65; −0.233) *p* < 0.001 * *p*(*BH*) < 0.001 *	−0.882 (−1.286; −0.479) *p* < 0.001 * *p*(*BH*) < 0.001 *	−0.296 (−0.692; 0.1) *p* = 0.143 *p*(*BH*) = 0.143	−0.358 (−0.575; −0.141) *p* = 0.001 * *p*(*BH*) = 0.01 *	−0.654 (−1.093; −0.215) *p* = 0.003 * *p*(*BH*) = 0.003 *
BDI	Training intensity	Overall sleep quality	−0.543 (−0.885; −0.202) *p* = 0.002 * *p*(*BH*) = 0.002 *	−0.339 (−0.571; −0.106) *p* = 0.004 * *p*(*BH*) = 0.017 *	−0.882 (−1.286; −0.479) *p* < 0.001 * *p*(*BH*) < 0.001 *	−0.396 (−0.763; −0.029) *p* = 0.034 * *p*(*BH*) = 0.039 *	−0.259 (−0.51; −0.007) *p* = 0.044 * *p*(*BH*) = 0.176	−0.654 (−1.093; −0.215) *p* = 0.003 * *p*(*BH*) = 0.003 *

* statistically significant.

## Data Availability

The data presented in this study are available on request from the corresponding author.

## Abbreviations

The following abbreviations are used in this manuscript:

BDI	Beck Depression Inventory
BMI	Body Mass Index
CES-D	Center for Epidemiologic Studies Depression Scale
CI	Confidence Interval
PA	Physical Activity
PSQI	Pittsburgh Sleep Quality Index
SEM	Structural Equation Modelling
